# An isocitrate lyase gene-deleted strain of *Nocardia seriolae* in live attenuated vaccine development against fish nocardiosis

**DOI:** 10.3389/fvets.2025.1664034

**Published:** 2025-10-31

**Authors:** Guanying Lin, Yan Li, Suying Hou, Yiming Wen, Xiaoya Lei, Ting Huang, Yuben Chen, Jianlin Chen, Liqun Xia

**Affiliations:** ^1^Guangdong Provincial Key Laboratory of Aquatic Animal Disease Control and Healthy Culture, Fisheries College of Guangdong Ocean University, Shenzhen Institute of Guangdong Ocean University, Zhanjiang, Guangdong, China; ^2^Guangxi Key Laboratory of Aquatic Genetic Breeding and Healthy Aquaculture, Guangxi Academy of Fishery Science, Nanning, Guangxi, China

**Keywords:** isocitrate lyase, attenuated vaccine, *Nocardia seriolae*, fish nocardiosis, relative percentage survival (RPS)

## Abstract

**Background:**

Fish nocardiosis caused by *Nocardia seriolae* is a severe bacterial disease in aquaculture, causing significant economic losses. The effectiveness of antibiotics in dealing with nocardiosis induced by *N. seriolae* is not satisfactory. NsICL is a potential virulence factor in *N. seriolae* based on its crucial role in the glyoxylate cycle, its identity as a secreted protein, and its established role in virulence and intracellular survival in homologous pathogens such as *Mycobacterium tuberculosis*. so, the NS-ΔICL deletion strain was constructed to investigate its function.

**Methods:**

The *NsICL* gene was knocked out via homologous recombination to generate the mutant strain NS-ΔICL. Comparative analysis of morphology, growth, and virulence was performed between the mutant and wild-type strains. The NS-ΔICL strain was used to immunize hybrid snakehead (*Channa maculata*♀ × *Channa argus*♂), with immune responses evaluated through non-specific immune parameters, specific antibodies, and expression levels of immune-related genes. Protective efficacy was assessed by challenge tests.

**Results:**

NS-ΔICL showed reduced virulence (LD_50_ = 2.60 × 10^5^ cfu/fish) compared to the wild-type strain (LD_50_ = 4.32 × 10^4^ cfu/fish). Immunized fish exhibited significantly higher levels of non-specific immune factors (AKP, ACP, POD, LZM) and specific IgM antibodies. Immune-related genes (*MHCI*α, *CD4, IL-8*) were upregulated in vaccinated fish. Relative percentage survival (RPS) was 77.03% in vaccinated fish after challenge tests, which indicating strong protective efficacy.

**Conclusion:**

The NS-ΔICL deletion strain was successfully constructed in this study. It can not only induce humoral and cellular immunity in fish but also effectively protect fish against *N. seriolae* infection. These results provide a foundation for the development of a live attenuated vaccine for treating fish nocardiosis.

## 1 Introduction

*Nocardia* are Gram-positive, catalase-positive, aerobic, non-motile, partially acid-fast and branched beaded and long filamentous bacteria in pleomorphic bacilli with unique features and complex characteristics. The genus *Nocardia* belongs to Class: Actinobacteria, Order: Actinomycetales, Suborder: Corynebacteriacea and Family: Nocardiae (1). It was first described as *Streptomyces salmonicida* in the diseased sockeye salmon (*Onchorhynchus nerka*) but then reclassified to the genus of *Nocardia* in 1949 (2). Up to now, approximately 113 *Nocardia* species have been identified from both aquatic and terrestrial environments (3, 4).

Fish nocardiosis is a chronic systemic granulomatous disease that causes significant impacts on both marine and freshwater aquaculture industries (5, 6). *Nocardia asteroides, Nocardia salmonicida, and N. seriolae* are pathogens that have been isolated from diseased fish affected by nocardiosis. For the past 30 years, *N. seriolae* has been recognized as the chief pathogen causing fish nocardiosis (7). The disease is characterized by the appearance of yellow or white granulomas, generally about 1–5 mm in diameter, inside the internal organs of the fish (1). Infected fish may also display nodules along the inner part of the operculum or on the gill filaments, fibromas in the abdominal cavity, and skin ulcers (6). According to the statistics, there are approximately 42 species, including marine fish as well as freshwater fish suffering from *N. seriolae* infection, such as silver pomfret (*Pampus argenteus*), large yellow croaker (*Larimichthys crocea*), yellowtail (*Seriola quinqueradiata*), largemouth bass (*Micropterus salmoides*), and hybrid snakehead (*Channa maculata*♀ × *Channa argus*♂) (7, 8).In recent years, the occurrence of nocardiosis has been increasing globally, leading to considerable economic losses, particularly in China (6, 9–11).

The effectiveness of antibiotics in dealing with nocardiosis induced by *N. seriolae* is not satisfactory (1). Antibiotics not only tend to cause environmental pollution and food safety issues, but also, as drug resistance builds up, they are unable to completely break through the nodule structure to exterminate *N. seriolae*. Therefore, using a vaccine is a good approach to control *N. seriolae* infection. Developing a healthy and useful way to prevent nocardiosis caused by *N. seriolae* is extremely important.

Vaccines usually offer effective immune protection by strengthening the host's immune reaction. While, Xie et al. found that inactivated vaccines of *N. seriolae* could not provide effective immune protection; it is assumed that the antigens might have lost their functions during the inactivation process (12). Hoang et al. discovered that subunit vaccines of hypoxia-responsive protein (rHRP1) and the resuscitation-promoting factor (tRPF) from *N. seriolae* showed 73.33% and 69.23% relative percentage survival (RPS) in largemouth bass, respectively (13). Nevertheless, recent studies propose that attenuated live vaccines might offer superior protection. For example, Li et al. obtained the U-20 attenuated strain through ultraviolet irradiation mutagenesis using LiCl- supplemented media. It was shown that vaccination with the U-20 strain offered an 89.65% RPS in hybrid snakehead (14). This emphasizes the potential of the attenuated vaccine to effectively stimulate the host's humoral and cellular immune responses by simulating a natural infection. The key to developing attenuated live vaccines is to obtain strains that are safe, have low toxicity, and possess good immunogenicity.

Isocitrate lyase (ICL) facilitates the conversion of isocitrate into succinate and glyoxylate, along with malate synthase, it ships two decarboxylation steps in the tricarboxylic acid cycle, directing the carbon flux toward the glyoxylate cycle (15). Studies have revealed that mice infected with wild-type *M. tuberculosis* died at 68–113 days, with an average of 88 days. In contrast, mice infected with *M. tuberculosis* with a deleted *ICL* gene survived for an average of 168 days, suggesting that *ICL* gene deletion reduced the virulence of *M. tuberculosis* (16). The *ICL* gene is crucial for the growth, survival, and virulence of *M. tuberculosis* in mice. *N. seriolae* ZJ0503 is a pathogenic strain isolated from diseased *Trachinotus ovatus* (golden pomfret) in our previous study (17). Upon analyzing the entire genome of *N. seriolae* ZJ0503, it was found that the open reading frame (ORF) 0653 encodes a homolog of ICL, which named as NsICL. Importantly, our study showed that NsICL is a possible virulence factor of *N. seriolae* (18). Therefore, in this study, we constructed a mutant strain (NS-ΔICL) by deleting the *NsICL* gene in wild-type strain *N. seriolae* ZJ0503. Moreover, we comprehensively evaluated the efficacy of the live vaccine NS-ΔICL in fish after immunization. This assessment covered histopathological analysis, quantification of serum non-specific immune parameters, detection of specific antibodies, profiling of immune-related gene expression, and calculation of RPS.

## 2 Materials and methods

### 2.1 Bioinformatics analysis

According to the whole genome sequence of *N. seriolae* ZJ0503 (accession no. NZ_JNCT01000022), sequence analysis was performed with the BLAST program using NCBI (http://www.ncbi.nlm.nih.gov/BLAST/). The amino acid sequence for NsICL was deduced, and the physical and chemical properties were predicted using ExPASy software (http://www.expasy.org/). The three-dimensional structure of the protein was predicted using the SWISS-MODEL website (https://swissmodel.expasy.org/) and modified by PyMOL software. Multiple sequence alignment analysis of amino acids from different bacterial species was performed using ClustalX 2.0 and GeneDoc software. The phylogenetic tree was constructed using the neighbor-joining method with MEGA 5.0 software.

### 2.2 Bacteria, plasmid, and fish

The wild-type strain of *N. seriolae* ZJ0503 was taken from a sick *Trachinotus ovatus* (golden pomfret) in Yangjiang, China in 2005. It is preserved in the Guangdong Provincial Engineering Research Center for Aquatic Animal Health Assessment and is cultivated using brain heart infusion medium (BHI). The deletion strain and complemented strain were constructed using the plasmid pRE112, which is preserved in our lab. The healthy hybrid snakehead were purchased from a fishery in Guangzhou, China, and the size was 13 ± 2 cm in length and 25 ± 5 g in weight. This study was approved by the Animal Research and Ethics Committee (AREC) of Guangdong Ocean University, China. All animal experiment operations were carried out following the university's regulations for animal experiments, and the animal facility was managed in line with the National Institutes of Health (NIH) guidelines for the care and use of laboratory animals (NIH Publication No. 8023, revised 1978).

### 2.3 Identification of secreted proteins

The extracellular products of *N. seriolae* ZJ0503 were obtained via the cellophane overlay method. *N. seriolae* ZJ0503 was cultured on optimized medium agar plates at 28 °C for 72 h to obtain well-grown bacterial colonies, and a single colony was prepared for bacterial suspension. Then, took 100 μl *N. seriolae* suspension and evenly spread it on the cellophane covered BHI plates, and cultivated at 28 °C for 3–5 days. *N. seriolae* cells grown on the cellophane sheet were stripped from the plates, and the extracellular products were subsequently washed off with sterilized PBS. The harvested suspension was centrifuged at 8,000 g at 4 °C for 20 min, and the supernatant containing extracellular products was filter sterilized with a 0.2 μm membrane filter. Then, the sterilized supernatant was transferred into a dialysis tube (3.5 kMW) and dialysed in ultrapure water at 4 °C for 16–24 h. During dialysis, the ultrapure water was changed 3–4 times. The purified supernatant was transferred into a centrifuge tube after dialysis and frozen at −80 °C. Finally, it was lyophilized using a vacuum freeze dryer to obtain the protein dry powder which was identified via shotgun mass spectrum (MS).

### 2.4 Construct deletion plasmid and complemented plasmid

This study is based on the whole genome sequence of *N. seriolae* ZJ0503 (accession no. NZ_JNCT01000022). PCR technology is used to amplify the upstream and downstream regions of the *NsICL* gene respectively. The primers used for this amplification were ICL-UF/ICL-UR for the upstream fragment and ICL-DF/ICL-DR for the downstream fragment (Supplementary Table 1). The two fragments were linked by overlapping PCR. The particular amplification procedure was as follows: 94 °C for 5 min, followed by 35 cycles of 94 °C for 30 s, 64 °C for 30 s, 72 °C for 30 s, and final elongation at 72 °C for 5 min. Next, the overlapping sequences were added to the pRE112 plasmid using Mlu I and Xba I restriction sites to create the deletion plasmid pRE112-ΔICL. Additionally, the *NsICL* gene was inserted into the pRE112 plasmid to obtain the complemented plasmid pRE112-cΔICL, and all recombinant plasmids were verified using specific primers (112-F/R) as detailed in Supplementary Table 1.

### 2.5 The construction of deletion strain and complemented strain

To obtain the deletion strain (NS-ΔICL) and the complementary strain (NS-cΔICL), the deletion plasmid pRE112-ΔICL and the complemented plasmid pRE112-cΔICL were transformed into the *N. seriolae* ZJ0503 by electroyransformation with a micro pulse (Etta biotech) according to the previously described method (19). This strategy utilized the sucrose-sensitivity conferred by the *sacB* gene present on the pRE112 vector, which allows for positive selection of double-crossover events and subsequent plasmid curing on media containing sucrose (20). First, 100 μL of *N. seriolae* cells (1 × 10^5^ cfu/mL) were mixed with 1 μg of the recombinant plasmid on ice for 30 min, then placed into a 96-well plate with 100 μL of mixed solution in each well. The electrotransformation method as follow: voltage 200 V, frequency 30, interval 1,000 ms, duration 60 ms. After electrotransformation, added 100 μL 28 °C preheated BHI liquid medium and incubated for 2 h at 28°C. The recovered bacteria were plated on BHI solid medium containing 20 mg/mL chloramphenicol for screening, and suspected positive clones were verified by PCR using primers 112F/R. Finally, the positive clones were inoculated into BHI liquid medium containing 10% sucrose to counter-select against bacteria that still retained the plasmid, based on the lethal effect of the *sacB* gene product in the presence of sucrose. After more than 30 generations of continuous culture, the stable hereditary strains NS-ΔICL and NS-cΔICL were obtained, and their stability was verified using ICL-F2/ICL-R2 primers.

### 2.6 Morphological observation of bacterial strains

The deletion strain NS-ΔICL and the wild-type strain were cultivated to the logarithmic growth phase, and 1 mL bacterial suspension of each strain was pipetted onto the slide to prepare a bacterial smear. The bacterial smears were subjected to Gram staining using a standard protocol. Briefly, the slides were stained with crystal purple for 1 min and subsequently rinsed with sterile water. Then, iodine solution was applied as a mordant for 1 min, followed by another rinse. Decolorization was performed by applying 95% ethanol and gently agitating the slide for approximately 1 min. Immediately after decolorization, the slide was washed with sterile water and blotted dry with absorbent paper. Finally, the samples were counterstained with safranin for 1 min, rinsed thoroughly with water, and air-dried prior to observation under an ordinary optical microscope.

### 2.7 The analysis of growth curve

To investigate the growth characters of NS-ΔICL, we monitored the growth rate of three strains (NS-ΔICL, NS-cΔICL and ZJ0503). Single colonies were picked from the plates and put into BHI liquid medium. Each strain was set up with three replicates, and the OD value of each strain was measured and recorded at 12 h intervals. The growth curves of three strains were established with OD value as ordinate and culture time as abscissa.

### 2.8 The pathogenicity of mutant strain

The challenge tests and fish survival experiments were conducted on hybrid snakehead (weight: 25 ± 5 g, length: 13 ± 2 cm) to evaluate the pathogenicity of NS-ΔICL and NS-cΔICL. To prepare for the challenge experiment, the hybrid snakehead were fed for 7 days. Subsequently, 30 fish per group (3 repetitions) were injected intraperitoneally with 100 μL of *N. seriolae* NS-ΔICL, NS-cΔICL, and ZJ0503 at concentrations of 104, 105, 106, 107, and 108 cfu/mL. In this study, we have verified that the differences in cfu/ml among the parent strain ZJ0503, mutant strain NS-ΔICL, and complemented strain NS-cΔICL under the same OD value are negligible, so the use of OD value to estimate bacterial concentration is reliable. After the challenge, the mortality rate was recorded daily for 14 days, and the median lethal bacterial dose (LD_50_) was calculated by probit analysis using the SPSS statistical software package.

### 2.9 The relative percentage survival of NS-ΔICL in fish

Healthy fish were randomly divided into 2 groups (designated as the NS-ΔICL group and the PBS group), with 3 repetitions per group, each containing 70 fish. Additionally, the immunized dosage of NS-ΔICL vaccine was determined by the aforementioned LD_50_ assay, and the LD_15_ (5.45 × 10^3^ cfu/fish) of the NS-ΔICL was chosen according to the calculations in Supplementary Tables 2, 3. The NS-ΔICL group was injected in the abdomen with 100 μL of NS-ΔICL suspension (5.45 × 10^3^ cfu/fish), while the PBS group was given 100 μL of sterile phosphate-buffered saline (PBS). All fish were fed under the condition of 28 ± 0.5 °C for 35 days post-vaccination (d.p.v.).

After 35 days of immunization with the attenuated vaccine NS-ΔICL or sterile phosphate-buffered saline (PBS), 2 groups (3 replicates/group) of surviving fish were challenged with 30 fish in each replicate. The bacterial suspension of wild-type strain *N. seriolae* ZJ0503 with a LD_50_ of 4.32 × 10^4^ cfu/fish was prepared according to the result of LD_50_ experiment. The 4.32 × 10^4^ cfu/fish of *N. seriolae* ZJ0503 was injected intraperitoneally into immunized fish at 100 μL/fish. Subsequently, mortality was recorded for 14 days after challenge. The relative percentage survival (RPS) was calculated using the following formula: RPS = [1 – (% mortality of immunized group/% mortality of control group)] × 100.

### 2.10 Detection of non-specific immune factors in serum

Following our previous study (21), at 1, 4, 7, 14, 21, 28, and 35 d.p.v., blood samples from 3 randomly selected fish in each group were collected from sterile syringes. After that, the professional protease detection kits provided by Nanjing Jiancheng Institute of Biological Engineering in China were used to carry out the activity levels of lysozyme (LZM), peroxidase (POD), alkaline phosphatase (AKP), and acid phosphatase (ACP) in serum samples.

### 2.11 Antibody level analysis

Similar to our previous study, the immunoglobulin M (IgM) titers in the serum were measured by enzyme-linked immunosorbent assay (ELISA). To expand, the *N. seriolae* bacterial suspension (1 × 108 cfu/mL) treated after 30 s of ultrasound was wrapped in a dose of 100 μL/well on the 96-well microplate, and carried out IgM detection on the 1, 4, 7, 14, 21, 28, and 35 days after immunity. Then, serum samples were added to each well and blocked with 2% BSA. Antibody combined with the antigen which was detected by using the rabbit anti-hybrid snakehead IgM antibody previously prepared by our lab. Microplates were incubated with goat anti-rabbit IgG HRP conjugate (BOSTER Biological Technology, China). The reaction was developed with a chromogenic reagent tetramethylbenzidine (Nanjing Jiancheng Bioengineering Institute, China) and stopped by 2.0 mol/L H_2_SO_4_. Absorbance at 450 nm was measured using a microplate reader (Bio-Rad, USA) (21).

### 2.12 Immune-related gene expression analysis

The kidney, spleen, and liver, which are immune-related organs in fish, were isolated from 3 randomly selected fish in each group at 1, 4, 7, 14, 21, 28, and 35 days after immunization. And the real-time fluorescence quantitative PCR technology was used to detect the expression levels of immune-related genes such as the main tissue compatibility complex class I α (*MHCI*α), differentiation cluster 4 (*CD4*) and Interleukin-8 (*IL-8*). Gene specific quantitative primes to detect aforementioned genes mRNA transcript expression are listed in Table 1. The qRT-PCR was carried out with the following program: 95 °C for 5 min, 95 °C for 15 s, 60 °C for 30 s, 40 cycles. All tests treat the β*-actin* gene as an internal reference gene, and each sample has 3 technical repetitions. The mRNA transcript levels of the immune-related genes were normalized to β*-actin* using the Ct 2^−ΔΔ*Ct*^ method.

**Table 1:** Genes and primer sequences used in the qRT-PCR assays.

**Genes**	**Forward primer (5^′^-3^′^)**	**Reverse primer (5^′^-3)**
*MHCIα*	TGCACTCATGGAAGGCATTTTA CAC	GGGTAGCCTCTGAGAA TGT
*CD4*	AATCTGTCTTCTGACCTCC AAC	CACCCATTTTCCGCTA TCT
*IL-8*	CCTGTGAAGGCATGG GTG	GCAGTGGGAGTTGGG AAG
*β-actin*	ACAATCAATACGGCTGCCA TGG	TTGGCATACAGGTCCTTA CTTCAGT

### 2.13 Statistics

All statistical analyses were generated in SPSS 21.0 software (IBM, Chicago, IL, USA). Data were presented as the means ± standard error of the mean (SEM) of triplicate vaccination. Differences between the means of the NS-ΔICL group and PBS control group were analyzed by Student's *t*-test. To account for multiple comparisons across time points, *p*-values were adjusted using the Holm-Bonferroni method. Results were considered significant at an adjusted *p* < 0.05.

## 3 Results

### 3.1 Bioinformatic analysis

Bioinformatic analysis revealed that the *NsICL* gene is 1,290 bp in length, and is predicted to encode a protein of 429 amino acids with a molecular weight of 46.72 kDa and an isoelectric point (pI) of 4.91. Domain prediction indicated that the protein belongs to the isocitrate lyase superfamily. Further analysis predicted that a region spanning nucleotides 56–362 bp in the *NsICL* gene encodes a functional domain characteristic of this family, and it is predicted that the amino acid sequence (189–194) of “KKCGHL“ constitutes a key part of the active site (Supplementary Figure 1). The three-dimensional structure of the NsICL protein was predicted using SWISS-MODEL (Supplementary Figure 2) and then compared with that of *M. tuberculosis*. The models showed significant overlap in their three-dimensional conformations and high structural similarity. This suggests that the function of NsICL may be similar to *M. tuberculosis*. Multiple sequence alignment demonstrated high conservation of ICL among *Nocardia, Rhodococcus*, and *Corynebacterium* (Supplementary Figure 3), while phylogenetic analysis clustered *N. seriolae* NsICL with other Actinobacterales homologs (Supplementary Figure 4). These results confirmed NsICL as a conserved metabolic enzyme and a potential virulence target, justifying its selection for gene knockout.

### 3.2 Identification of NsICL as a secreted protein

The extracellular products of *N. seriolae* were obtained, and the secreted proteins were identified using shotgun MS. Results showed the peptide sequences of NsICL (VEGDTSVANWLAPIVADAEAGFGGALNAYELQK) were detected with confidence greater than or equal to 99%, which proved that NsICL was a secreted protein of *N. seriolae*.

### 3.3 Construction, characterization, and pathogenicity of mutant strains

#### 3.3.1 Construction of the deletion plasmid, complemented plasmid, and mutant strain

Using designed primers based on the genome data of *N. seriolae* ZJ0503, the upstream and downstream fragments of the *NsICL* gene were successfully cloned, each about 506 bp (Supplementary Figure 5A). Additionally, the overlapping PCR fragment of 1,012 bp (Supplementary Figure 5B) and the complementing fragment of 2,302 bp (Supplementary Figure 5C) were also successfully cloned. The above cloned products were confirmed to be correct by sequencing. The deletion plasmid (pRE112-ΔICL) and the complemented plasmid (pRE112-cΔICL) were successfully constructed. The positive clones were screened by PCR using the primer 112-F/112-R to detect the plasmids pRE112-ΔICL and pRE112-cΔICL. The results show that both the deletion strains and the complemented strains can amplify a fragment of about 262 bp of pRE-112, except the ZJ0503 (Supplementary Figure 6A). The pRE112-ΔICL is a suicide plasmid that can be eliminated by increasing the concentration of sucrose in the medium, and the sucrose treated NS-ΔICL did not contain the 262 bp fragment, while it still existed in the untreated NS-ΔICL (Supplementary Figure 6B). The *NsICL* can be amplified in complemented strain NS-cΔICL and wild-type strain ZJ0503 with primer ICL-F2/ICL-R2, but it cannot be amplified in the deletion strain NS-ΔICL. The successful construction of NS-ΔICL and NS-cΔICL has been verified in Supplementary Figure 6C. After the 30th generation of continuous culture, the *NsICL* gene was detected to verify the genetic stability of NS-ΔICL and NS-cΔICL. The fragment of *NsICL* cannot be amplified with ICL-F2/ICL-R2 in NS-ΔICL, but it can be successfully amplified in NS-cΔICL and ZJ0503 (Supplementary Figure 6D).

#### 3.3.2 Morphology and growth characteristics of different strains

The morphology and growth differences between the NS-ΔICL, NS-cΔICL and ZJ0503 were studied. The results of gram staining showed that both NS-ΔICL and ZJ0503 were filamentous, and there were no significant differences in morphology between the two strains (Supplementary Figure 7). In addition, under the same culture conditions, the OD values of the three *N. seriolae* strains were measured every 12 h, and the growth curves were plotted. It was found that there was no difference in the growth of the three strains; they all entered the logarithmic growth phase at 48 h and the plateau phase at 120 h (Supplementary Figure 8).

#### 3.3.3 Pathogenicity of mutant strains (LD_50_ determination)

To evaluate the pathogenicity of NS-ΔICL and NS-cΔICL, challenge tests were performed on hybrid snakehead (weight: 25 ± 5 g, length: 13 ± 2 cm) following the method described in Section 2.7 The median lethal dose (LD_50_) of NS-ΔICL, NS-cΔICL, and ZJ0503 were calculated as 2.60 × 10^5^ cfu/fish, 4.26 × 10^4^ cfu/fish, and 4.32 × 10^4^ cfu/fish, respectively. This indicates that the virulence of *N. seriolae* decreased with the deletion of *NsICL* gene. All the diseased fish showed the clinical symptoms of nocardiosis, such as a large number of white nodules on the immune organs, damage to the body surface, ulceration, bleeding, and abdominal distension. Additionally, the bacteria were re-isolated from the nodules of diseased fish and identified as *N. seriolae* by PCR and sequencing.

### 3.4 Analysis of immune parameters in serum

**Figure 1:**
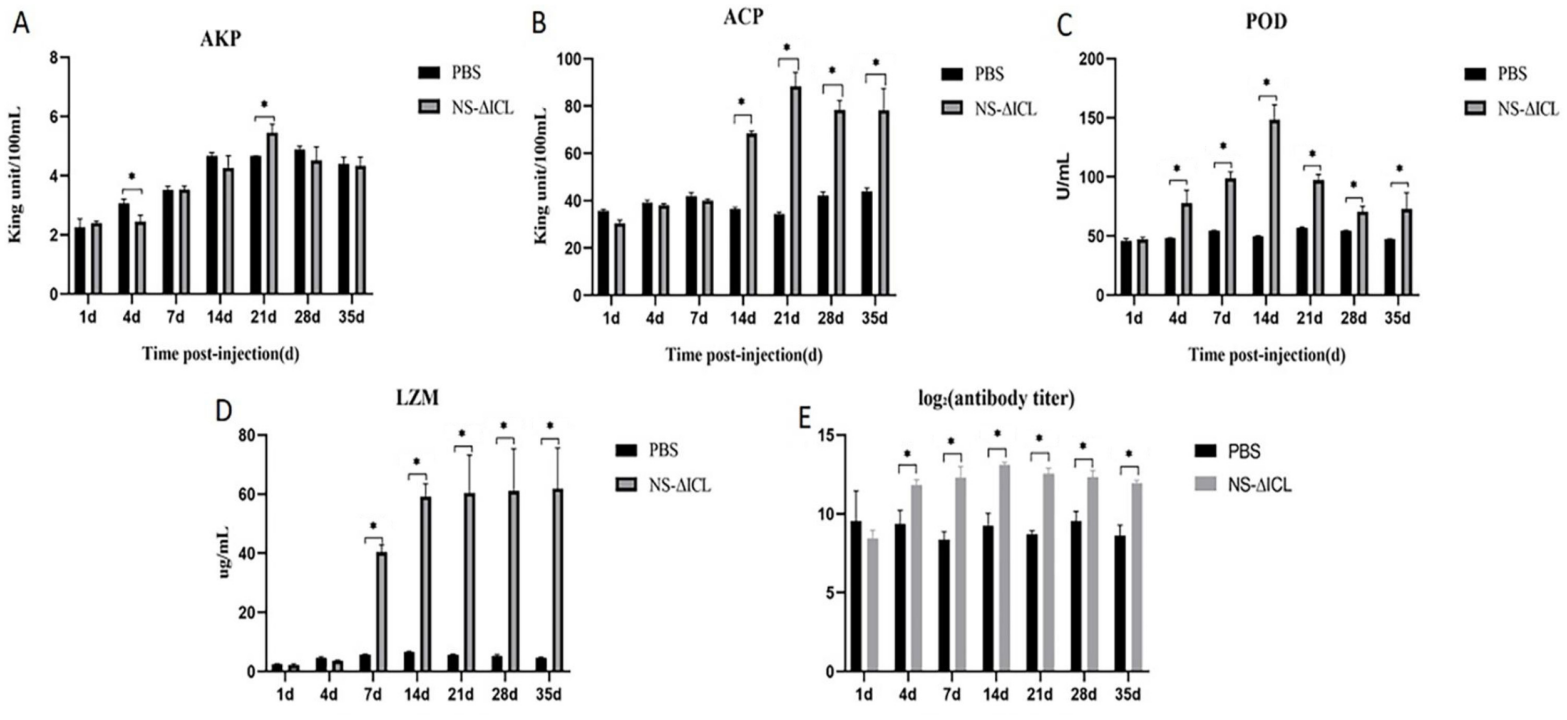
Immune response of hybrid snakehead after immunization with the attenuated NS-ΔICL vaccine. **(A)** Alkaline phosphatase (AKP), **(B)** acid phosphatase (ACP), **(C)** peroxidase (POD), **(D)** lysozyme (LZM) activities, and **(E)** specific IgM antibody levels in serum at 1, 4, 7, 14, 21, 28, and 35 days post-vaccination (d.p.v.). Data are presented as the mean ± SD (*N* = 3 biological replicates; serum samples from three individual fish per group per time point, with each sample measured in technical triplicate). Asterisks indicate significant differences between the NS-ΔICL group and the PBS control group (*p < 0.05).

After immunization with the NS-ΔICL vaccine, serum samples from fish in the PBS group and the NS-ΔICL group were collected at 1, 4, 7, 14, 21, 28, and 35 d.p.v., respectively. At 21 d.p.v., AKP activity in serum stimulated by NS-ΔICL was significantly higher than that in the PBS group (Figure 1A). During 14–35 d.p.v., the serum ACP activity of NS-ΔICL group was significantly higher than PBS group and peaked at 21 d.p.v. (Figure 1B). During 4–35 d.p.v., POD activity in the NS-ΔICL group was significantly higher than that of PBS group (Figure 1C), reaching the highest level at 14 d.p.v. During 7–35 d.p.v., LZM activity in the serum stimulated by NS-ΔICL was significantly higher than that in the PBS group (Figure 1D). The specific antibodies IgM were detected by ELISA at 1, 4, 7, 14, 21, 28, and 35 d.p.v., respectively. The results showed that IgM levels were significantly higher in the NS-ΔICL group than in the PBS group during the period of 4~35 d.p.v. and peaked at 14 d.p.v. (Figure 1E).

### 3.5 Analysis of immune-related genes

**Figure 2:**
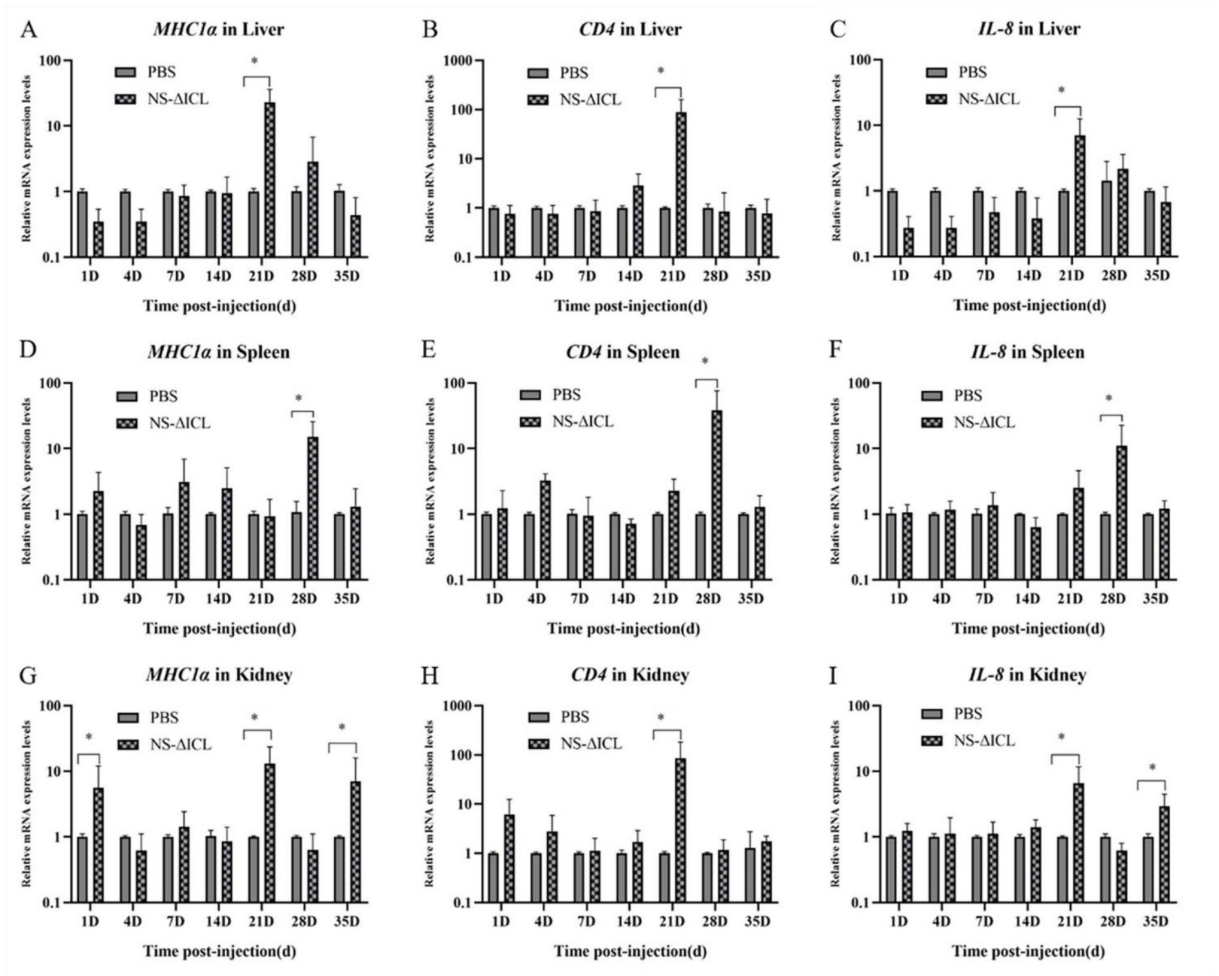
qRT-PCR analysis of the expression of immune-related genes in liver **(A–C)**, spleen **(D–F)**, and kidney **(G–I)**. The data were shown as the expression of *MHCI*α, *CD4* and *IL-8*. The mRNA expression level of each gene was normalized by β*-actin*. Bars represented the mean relative expression of three biological replicates (*N* = 3), and error bars represented standard deviation. The asterisk indicated the significant difference among different groups (*p < 0.05).

At the transcriptional level, we further analyzed the expression of three immune-related genes *(MHCI*α*, CD4* and *IL-8*) in the liver, spleen, and kidney by qRT-PCR. The results revealed that the expression of the *MHCI*α gene reached its peak at 21 d.p.v. in the liver (Figure 2A) and kidney (Figure 2G), while it peaked at 28 d.p.v. in the spleen (Figure 2D). The expression patterns of the *CD4* and *IL-8* genes were similar in the liver and kidney; they both peaked at 21 d.p.v. (Figures 2B, C, H, I). The expression patterns of the *CD4* and *IL-8* genes in the spleen were also similar, both peaking at 28 d.p.v. (Figures 2E, F). It is interesting to note that the expression of the *MHCI*α and *IL-8* genes in the kidney not only peaked at 21 d.p.v., but was also significantly higher at 35 d.p.v. than in the PBS group (Figures 2C, I).

### 3.6 The efficacy of the vaccine on *N. seriolae* infection

**Figure 3:**
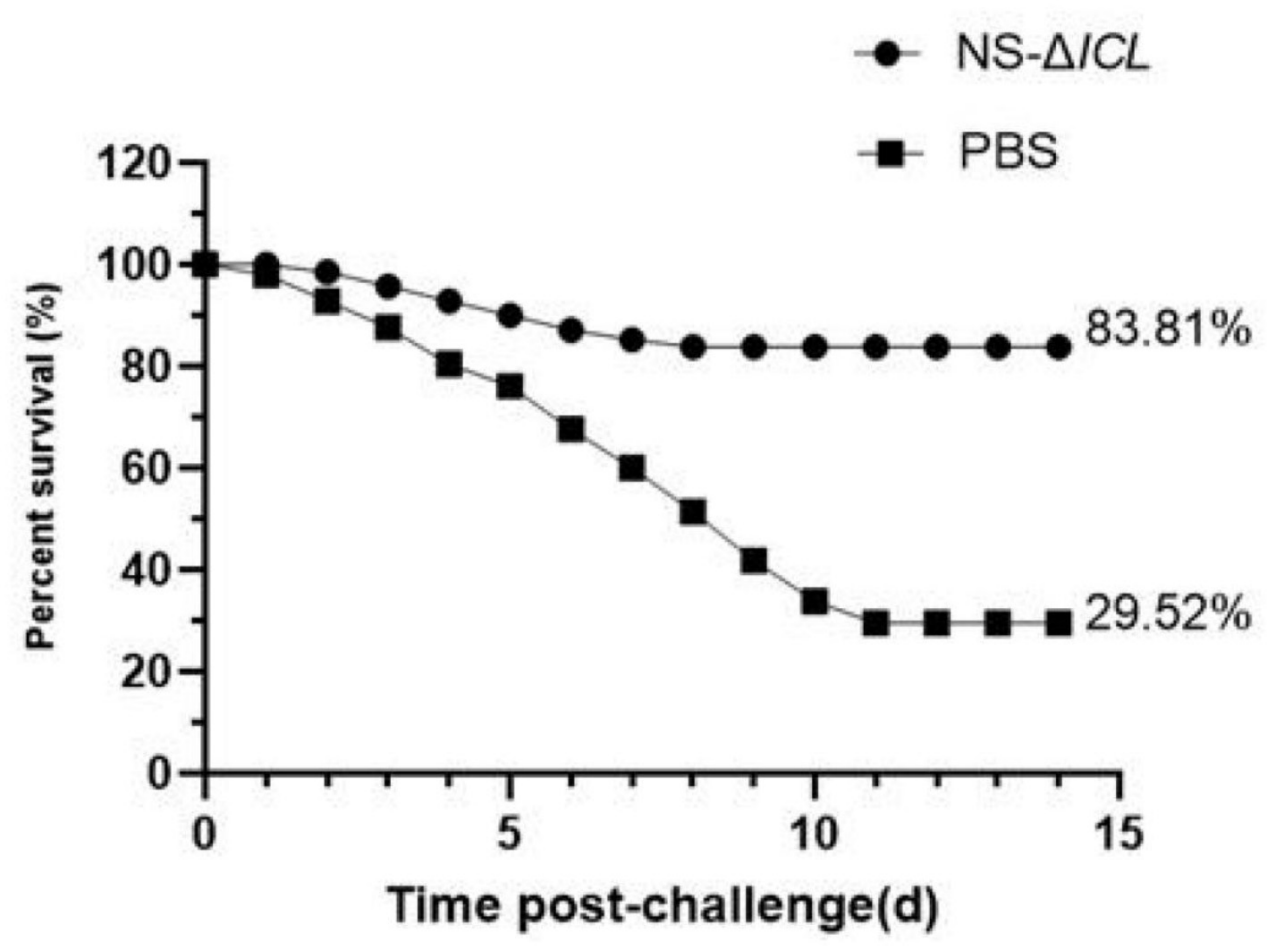
The attenuated vaccine NS-ΔICL provided effective protection against *Nocardia seriolae* challenge in hybrid snakehead. Fish were immunized with NS-ΔICL or PBS (control) and then challenged intraperitoneally at 35 days post-vaccination (d.p.v.) with the wild-type strain *N. seriolae* ZJ0503 at a dose of 4.32 × 104 cfu/fish. Cumulative survival was monitored for 14 days post-challenge. The final survival rate in the NS-ΔICL immunized group (83.81%) was significantly higher than that in the PBS control group (29.52%).

After 35 days of immunization, the cumulation mortality rates of NS-ΔICL and PBS were 10.19% and 1.42%, respectively. A challenge experiment with wild-type strain *N. seriolae* ZJ0503 was conducted at 35 d.p.v. to detect the vaccine efficacy. After the challenge, the cumulative survival rates of fish in NS-ΔICL group and PBS group were measured (Figure 3). The survival rates of fish in the PBS group and the NS-ΔICL group were 29.52% and 83.81%, respectively. The RPS of fish vaccinated with NS-ΔICL was 77.03%, indicating that the NS-ΔICL vaccine provided a good immune protective effect against *N. seriolae* infection. All the symptoms observed on diseased fish were consistent with those reported for fish nocardiosis.

## 4 Discussion

*N. seriolae* has caused worldwide epidemic of fish nocardiosis, posing a severe threat to both marine and freshwater aquaculture industries (22). As a facultative intracellular bacterium, during the process of infection, *N. seriolae* can be engulfed by phagocytes in the immune organs of fish, such as the spleen and head kidney (23). Concurrently, granulomatous structures are formed, which render antibiotics and other chemical drugs ineffective, as they struggle to penetrate these structures and completely eliminate *N. seriolae* (1). Currently, there is a lack of effective drugs and vaccines for the treatment and prevention of *N. seriolae* infection.

Previous studies have shown that bacterial-secreted proteins play a significant role in helping pathogens evade host immune responses, as well as promoting their survival, proliferation, and pathogenicity. For example, the secreted proteins of *Nocardia asteroids* and *Nocardia cyriacigeogica* can induce apoptosis in host cells, which is an important mechanism underlying their pathogenicity (24, 25). Our research identified NsICL as a secreted virulence factor of *N. seriolae* (18), and bioinformatics analysis in this study further confirmed its potential as a vaccine target. The *NsICL* gene encodes a 429-amino-acid protein belonging to the isocitrate lyase superfamily, with a conserved active site “KKCGHL“ (aa 189–194) and high three-dimensional structural similarity to the ICL protein of *M. tuberculosis* (Supplementary Figures 1, 2). Additionally, ICL sequences are highly conserved across *Actinobacterales* genera such as *Nocardia, Rhodococcus*, and *Corynebacterium* (Supplementary Figure 3), suggesting a conserved functional role in these pathogens. These characteristics make NsICL a suitable candidate for the development of an attenuated vaccine.

Gene knockout is one of the most powerful tools for researching gene function. Knockout techniques include homologous recombination, random insertion mutation, and the most current CRISPR/Cas system-mediated knockout techniques (26, 27). Among these, gene knockout via homologous recombination is the major technique employed to investigate the function of bacterial genes (28). In this study, the deletion strain NS-ΔICL was constructed by homologous recombination. The purpose was to study the influence of NsICL on the virulence of *N. seriolae*. The results indicated that there was no disparity in mycelial morphology between NS-ΔICL and the wild-type strain. Moreover, the growth curves of the three *N. seriolae* strains (NS-ΔICL, NS-cΔICL, and ZJ0503) in liquid medium were nearly identical, all entering the logarithmic growth phase at 48 h and the plateau phase at 120 h. These findings suggest that the deletion of the *NsICL* gene does not significantly affect the morphology or *in vitro* growth of *N. seriolae*. This is consistent with the study by McKinney et al., who found that the growth of the *ICL* deletion strain of *M. tuberculosis* was not significantly different from that of the wild-type strain, indicating that *ICL* deletion has no impact on the *in vitro* growth of these intracellular pathogens (16).

In the study of bacterial pathogenicity, LD_50_ is the curcial index for measuring bacterial pathogenicity, as changes in the LD_50_ of knockout strains can determine whether a specific gene is an important virulence factor (29). For instance, in *Aeromonas veronii*, the LD_50_ of an nucleoside diphosphate kinase (NDK) knockout strain was significantly higher than that of the wild-type strain. This demonstrating NDK's significance in virulence (30). In contrast, in *Edwardsiella tarda*, the LD_50_ of a phospholipase D superfamily protein gene knockout strain showed no obvious difference, suggesting a minimal impact on pathogenicity (31). In this research, the LD_50_ of the NS-ΔICL was approximately 10 times higher than that of the wild-type strain and the complemented strain. This clearly indicates that the *NsICL* gene is closely related to the virulence of *N. seriolae*.

Notably, the role of the *ICL* gene in pathogenicity varies across different pathogens. The *ICL* gene signifcantly affects the pathogenicity of *M. tuberculosis*. Mice infection experiments showed that the wild-type strain caused a 100% mortality rate, while all mice infected with the *ICL* gene-deficient strain survived (16). In contrast, the NS-ΔICL only showed a moderate reduction in virulence, but *M. tuberculosis* ICL-deficient strains had a complete loss of pathogenicity. This difference may be attributed to the complexity of the virulence system of *N. seriolae*, which is controlled by multiple virulence factors, and NsICL is just one of them. Our previous studies support this hypothesis: the LD_50_ of the mutant strain NS-ΔAld was approximately seven times higher than that of the wild-type *N. seriolae* (32), and the LD_50_ of the mutant strain NS-ΔGlu was also around seven times higher (19). Additionally, using subclinical doses for fish immunization has been proven a reasonable strategy in numerous studies, as subclinical dosages can efficiently activate the immune system without causing unwanted reactions, ensuring the safety of fish and enhancing the effectiveness of the immune response (33–36). Therefore, in future studies, constructing multiple virulence gene deletion strains may be a viable approach to obtain low-virulence or even avirulent strains for the development of live vaccines, offering a safer and more effective method for immunizing fish against *N. seriolae*.

Teleost fish, as lower vertebrates, mainly depend on innate immunity to get rid of invasive pathogens (21). The innate immune system includes several non-specific immune enzymes, such as AKP, ACP, LZM, and POD, which serve as the front line of defense. AKP and ACP enhance the phagocytic and degradative actions of phagocytes against pathogenic microorganisms by modifying their surface structures (37). LZM destroys bacterial cell walls and activates the complement system. It also synthesizes hypochlorous acid through reactive oxygen species and destroys pathogens by peroxidase (POD) (38). Superoxide dismutase (SOD) is in charge of scavenging excess superoxide free radicals to protect host cells from oxidative damage (39). In humoral immunity, the production of antibodies is crucial for specific immune responses and preventing bacterial infections. In teleost fish, IgM is the basic component of the humoral immune response and is usually regarded as the main antibody during the primary immune response (40).Our study showed that immunization with the attenuated vaccine NS-ΔICL led to considerable increases in serum non-specific immune parameters, such as the activities of LZM, POD, ACP, and AKP. Also, the titers of specific antibodies (IgM) were elevated. These results demonstrate that the attenuated vaccine NS-ΔICL can effectively activate the host's innate and humoral immune responses, thereby enhancing the fish's ability to resist *N. seriolae* infection.

The stimulation of cell-mediated immunity is another vital sign of vaccine efficacy. We therefore analyzed the expression levels of three immune-related genes (*MHCI*α, *IL-8*, and *CD4*) in the liver, spleen, and kidney of fish after immunization with NS-ΔICL. *MHCI*α primarily serves to present endogenous antigenic peptides to CD8^+^ T cells. This activates cytotoxic T cells, enabling them to recognize and eliminate pathogen-infected host cells. CD4^+^ T cells act as T helper cells to facilitate the activation of the immune response in teleost fish (41). *IL-8* is essential for fish innate immunity, as it regulates inflammatory responses and directs the movement of immune cells (42). The results showed that compared with the control group, the expression of these immune-related genes was significantly increased. *MHCI*α peaked at 21 d.p.v. in the liver and kidney and at 28 d.p.v. in the spleen; *CD4* and *IL-8* peaked at 21 d.p.v. in the liver and kidney and at 28 d.p.v. in the spleen (Figure 2). Notably, the expression of *MHCI*α and *IL-8* in the kidney was also significantly higher at 35 d.p.v. than in the PBS group, suggesting the induction of long-term cellular immune memory. This indicates that NS-ΔICL has the potential to induce robust cellular immune responses in fish, providing new insights for the investigation of attenuated vaccines. Addition, When challenged with the wild-type virulent strain ZJ0503 (LD_50_ = 4.32 × 10^4^ cfu/fish), the NS-ΔICL immunized group achieved a survival rate of 83.81%, whereas the PBS control group only reached 29.52%. Calculations yielded a relative percentage survival (RPS) of 77.03% for the vaccine group, confirming robust protective efficacy against *N. seriolae* infection.

Despite these promising results, this study has several limitations. First, the immunization route used was intraperitoneal injection, which is not suitable for large-scale aquaculture due to its high labor cost and stress on fish. Oral administration and immersion are more practical immunization routes in aquaculture, and future studies should explore these routes to evaluate the immunogenic efficacy and feasibility of the NS-ΔICL vaccine. Second, we only evaluated the vaccine's efficacy in hybrid snakehead, but *N. seriolae* can infect approximately 42 fish species, including silver pomfret, large yellow croaker, and largemouth bass (7). It is necessary to verify the cross-protective effect of NS-ΔICL in other susceptible fish species. Third, the virulence reduction of NS-ΔICL is moderate, and constructing multi-gene deletion strains may further reduce virulence while retaining immunogenicity, improving vaccine safety.

In conclusion, the NS-ΔICL deletion strain was successfully constructed in this study. It exhibits reduced virulence but unchanged morphology and growth characteristics, can induce both humoral and cellular immunity in fish, and provides effective protection against *N. seriolae* infection with an RPS of 77.03%. These results provide a solid foundation for the development of a live attenuated vaccine for the prevention and control of fish nocardiosis. For pathogens like *N. seriolae* that exhibit multi-factor synergistic virulence, constructing multiple gene deletion strains and optimizing immunization routes will be key directions for future vaccine development.

## Data Availability

The original contributions presented in the study are included in the article/Supplementary material, further inquiries can be directed to the corresponding author.
