# Supplementary material for: An isocitrate lyase gene-deleted strain of Nocardia seriolae in live attenuated vaccine development against fish nocardiosis
Source: Front Vet Sci. 2025 Oct 31;12:1664034. doi: 10.3389/fvets.2025.1664034 (PMC12615200; doi:10.3389/fvets.2025.1664034)
Supplement: Supplementary file 1 [file Data_Sheet_1.pdf]

Supplementary Figure:

```
1       ATGTCGAACGTCGGCACCCCGAAGACCGCTGCGGAACTCCAGAAGGATTGGGACACCAAC
1        M  S  N  V  G  T  P  K  T  A  A  E  L  Q  K  D  W  D  T  N
61      CCCCGCTGGAAGGGCGTCACCCGCAACTACACCGCCGATCAGGTTGTGAAGCTCCAGGGC
21       P  R  W  K  G  V  T  R  N  Y  T  A  D  Q  V  V  K  L  Q  G
121     ACCGTCGTCGAGGAGCACACCCTCGCTCGTCGTGGCGCCGAGATCCTCTGGGATCTCGTC
41       T  V  V  E  E  H  T  L  A  R  R  G  A  E  I  L  W  D  L  V
181     AACAACGAGGACTACATCAACTCCCTGGGCGCCCTCACCGGCAACCAGGCCGTGCAGCAG
61       N  N  E  D  Y  I  N  S  L  G  A  L  T  G  N  Q  A  V  Q  Q
241     GTCCGCGCGGGCCTGAAGGCCATCTACCTGTCGGGTTGGCAGGTCGCCGGTGACGCGAAC
81       V  R  A  G  L  K  A  I  Y  L  S  G  W  Q  V  A  G  D  A  N
301     CTGTCCGGCCACACCTACCCGGACCAGTCGCTGTACCCGGCCAACTCGGTGCCCGCGGTC
101      L  S  G  H  T  Y  P  D  Q  S  L  Y  P  A  N  S  V  P  A  V
361     GTGCGTCGCATCAACAACGCGCTGCTGCGCGCCGACGAGATCGCCAAGGTCGAGGGTGAC
121      V  R  R  I  N  N  A  L  L  R  A  D  E  I  A  K  V  E  G  D
421     ACCTCGGTCGCCAACTGGCTGGCCCCGATCGTCGCCGACGCCGAGGCCGGCTTCGGTGGC
141      T  S  V  A  N  W  L  A  P  I  V  A  D  A  E  A  G  F  G  G
481     GCGCTGAACGCCTACGAGCTGCAGAAGGCCATGATCGCCTCCGGCGCGGCCGGCGTGCAC
161      A  L  N  A  Y  E  L  Q  K  A  M  I  A  S  G  A  A  G  V  H
541     TGGGAGGACCAGCTGGCCTCCGAGAAGAAGTGCGGCCACCTGGGCGGCAAGGTGCTCATC
181      W  E  D  Q  L  A  S  E  K  K  C  G  H  L  G  K  V  L  I
601     CCCACCCAGCAGCACATCCGCACCCTGACCTCCGCGCGTCTGGCCGCCGACGTCGCCGAC
201      P  T  Q  Q  H  I  R  T  L  T  S  A  R  L  A  A  D  V  A  D
661     GTGCCGTCGGTGATCATCGCCCGCACCGACGCCGAGGCCGCCACCCTCATCACCTCCGAT
221      V  P  S  V  I  I  A  R  T  D  A  E  A  A  T  L  I  T  S  D
721     GTGGACGAGCGCGACCGTGAGTTCCTGGACGGCACCCGCACCGCCGAGGGCTTCTTCGGT
241      V  D  E  R  D  R  E  F  L  D  G  T  R  T  A  E  G  F  F  G
781     ACCCGCAACGGCATCGACCCCTGCATCGCGCGTGCCAAGGCCTACGCCCCCTACGCCGAC
261      T  R  N  G  I  D  P  C  I  A  R  A  K  A  Y  A  P  Y  A  D
841     CTCATCTGGATGGAGACCGGCGTGCCGGACCTCGAGGTCGCGCGCAAGTTCGCCGAGTCG
281      L  I  W  M  E  T  G  V  P  D  L  E  V  A  R  K  F  A  E  S
901     GTTCGCAGCGAGTTCCCGGACCAGCTGCTGGCCTACAACTGCTCGCCGTCCTTCAACTGG
301      V  R  S  E  F  P  D  Q  L  L  A  Y  N  C  S  P  S  F  N  W
961     AAGGCGCACCTGGACGACGCGACCATCGCGAAGTTCCAGCGCGAGCTGGGCGCCATGGGC
321      K  A  H  L  D  D  A  T  I  A  K  F  Q  R  E  L  G  A  M  G
1021    TTCAAGTTCCAGTTCATCACCCTGGCCGGCTTCCACTCGCTGAACTACGGCATGTTCGAC
341      F  K  F  Q  F  I  T  L  A  G  F  H  S  L  N  Y  G  M  F  D
1081    CTGGCCTACGGCTACGCCCGCGAGGGCATGACCGCCTTCGTCGACCTGCAGGAGCGCGAG
361      L  A  Y  G  Y  A  R  E  G  M  T  A  F  V  D  L  Q  E  R  E
1141    TTCAAGGCCGCCGCCGAGCGTGGCTTCACCGCCGTCAAGCACCAGCGTGAGGTCGGCGCC
381      F  K  A  A  A  E  R  G  F  T  A  V  K  H  Q  R  E  V  G  A
1201    GGCTACTTCGACACCATCGCGACCACCGTCGACCCCAACACCAGCACCGCTGCGCTGAAG
401      G  Y  F  D  T  I  A  T  T  V  D  P  N  T  S  T  A  A  L  K
1261    GGCTCGACCGAAGAGGGCCAGTTCCACTGA
421      G  S  T  E  E  G  Q  F  H  *
```

Figure 1 The full-length nucleotide and deduced amino acid sequence of NsICL gene

the sequence in bold (aa 56 to 362) indicates isocitrate lyase prodomain , the underlined sequence (aa 189 to 194) represents the active site, "*" means the terminator

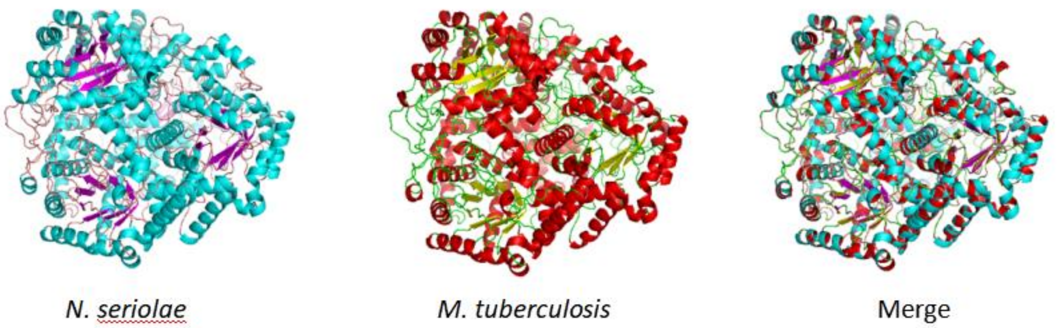

Figure 2 Prediction of the three-dimensional structure of NsICL protein from *N. seriolae* (Left), *M. tuberculosis* (Middle) and the overlapped three-dimensional structure of protein from them (Right).

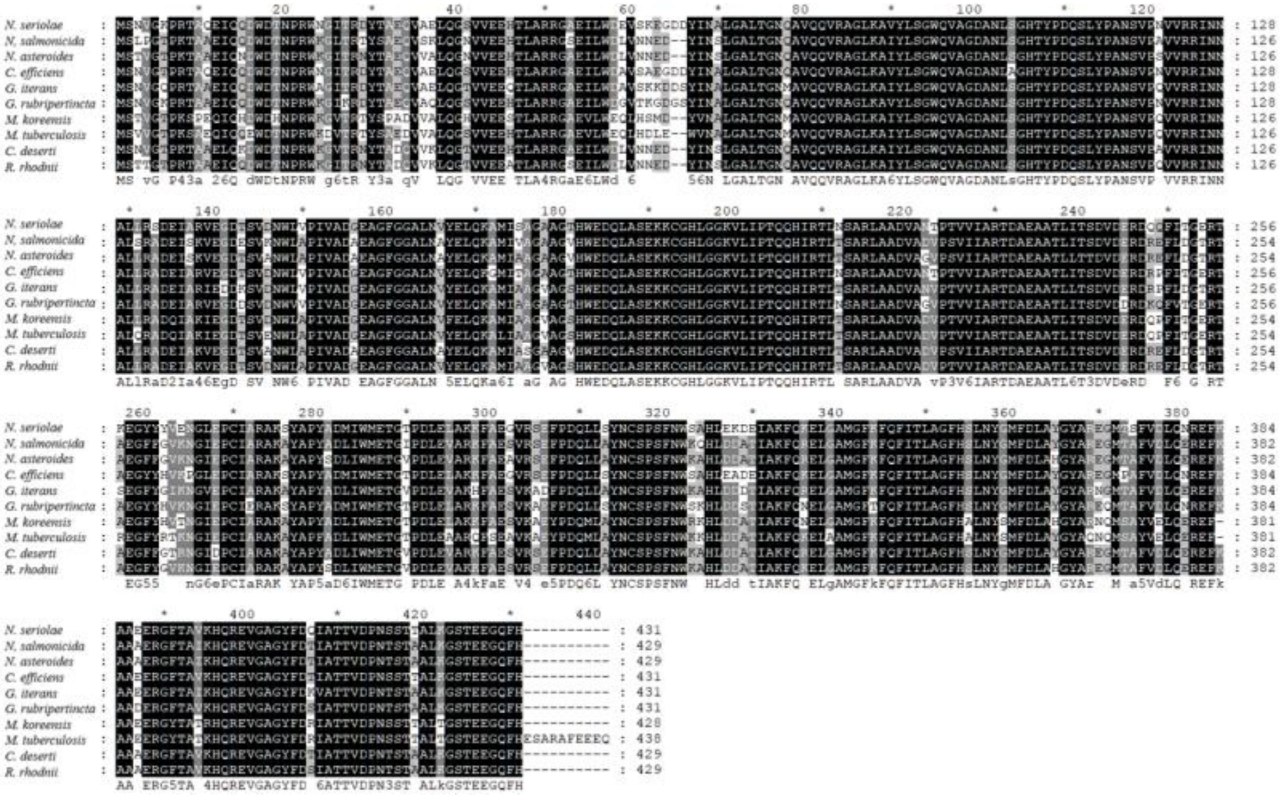

Figure 3 Multisequence alignment of isocitrate lyase homolog in Actinobacterales

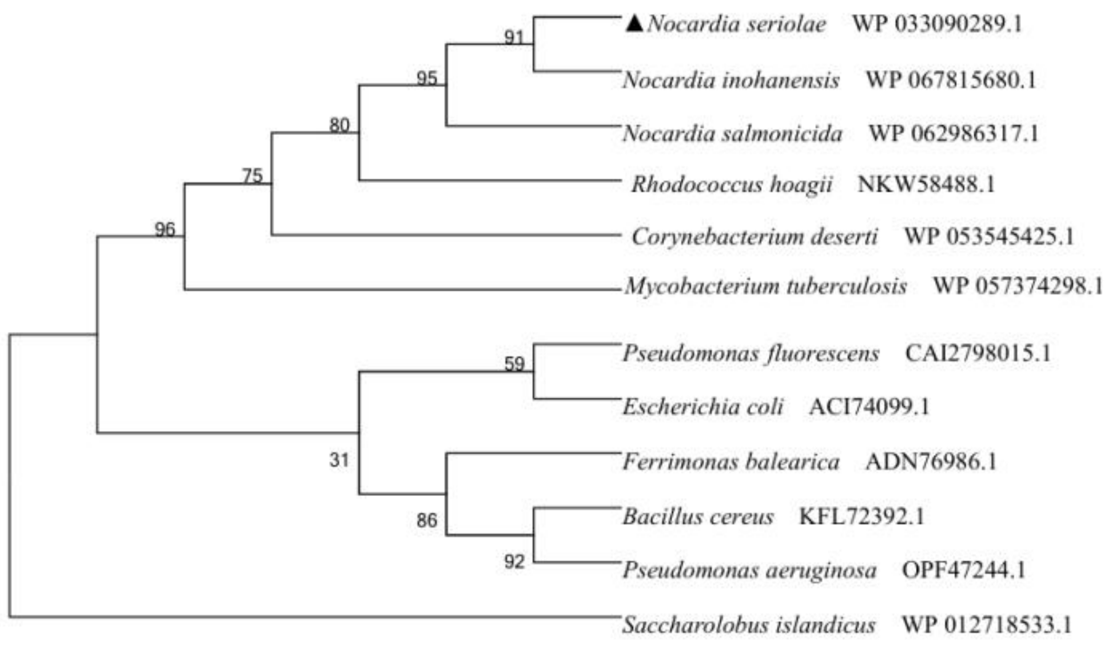

Figure 4 Evolutionary tree constructed by amino acid sequences of isocitrate lyase homologs.

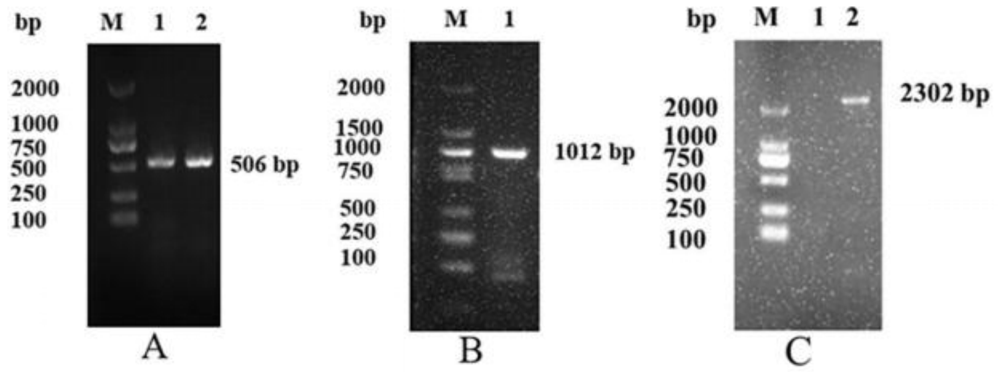

Figure 5 Gene cloning for from *N. seriolae* ZJ0503

A: *NsICL* gene upstream and downstream fragments M: DL2000 Marker, 1: Upstream fragments, 2: Downstream fragments; B: Overlapping PCR fragments M: DL2000 Marker; 1: Overlapping PCR fragments; C: *NsICL* gene complementing fragments M: DL2000 Marker, 1: Blank control, 2: Complementing fragments

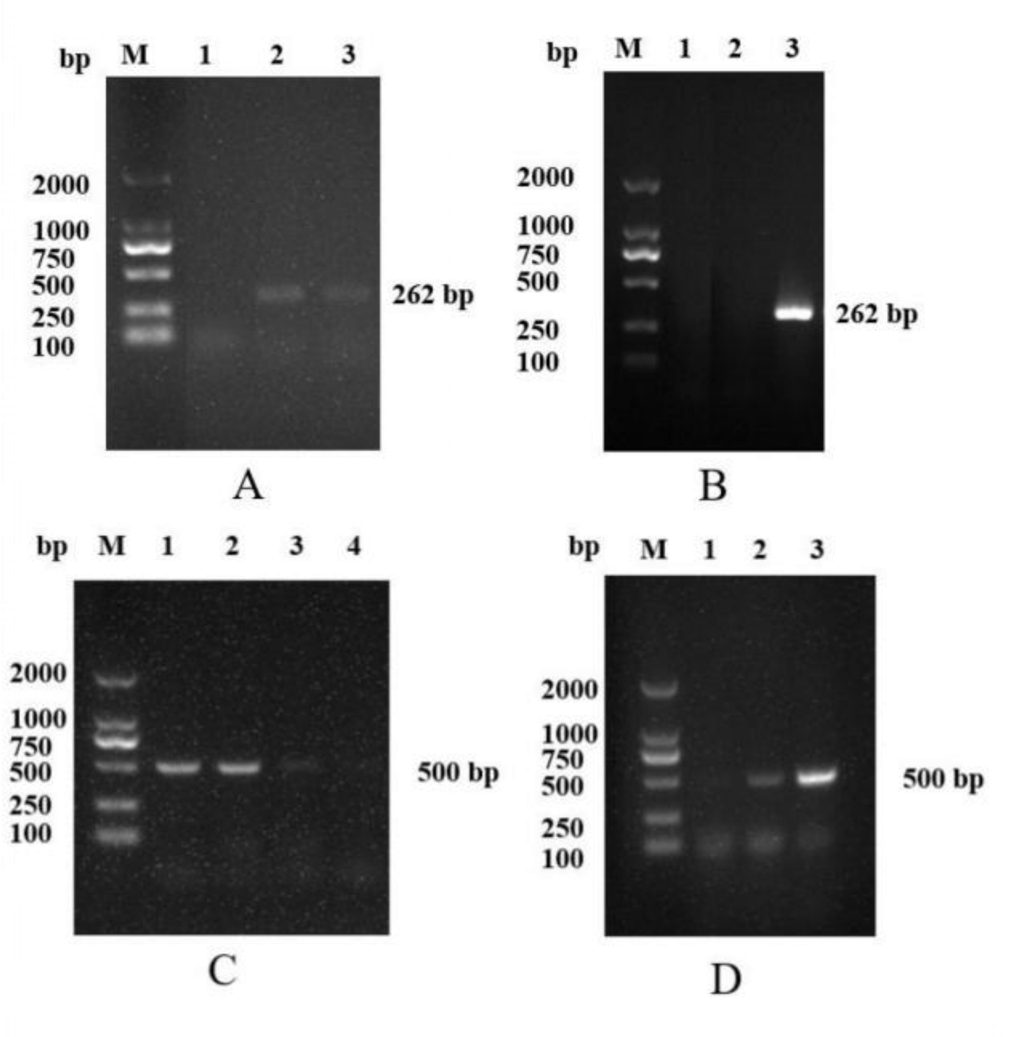

Figure 6 Verification of the deletion strain (NS-ΔICL) and the complemented strain(NS-cΔICL).

(A) PCR verification of the presence of the pRE112 plasmid in transformants using primers 112-F/112-R. The suicide plasmid pRE112 should be present in both the deletion (NS-ΔICL) and complemented (NS-cΔICL) strains prior to sucrose curing, yielding 262 bp band. As expected, this band was amplified in lanes 2 and 3 but not in the wild-type ZJ0503 (lane 1), which lacks the plasmid.

(B) Verification of plasmid elimination by sucrose counter-selection. After sucrose treatment, the pRE112 plasmid should be lost from the strains, resulting in the disappearance of the 262 bp band. The band was absent in the sucrose-treated NS-ΔICL (lane 1) and NS-cΔICL (lane 2), confirming successful plasmid curing, unlike the positive control (lane 3).

(C) PCR verification of the *NsICL* gene locus using primers ICL-F2/ICL-R2. Successful gene deletion should result in the loss of the *NsICL* amplification band in the NS-ΔICL mutant. As expected, the band was present in the wild-type (lane 1) and the complemented strain (lane 2, which restored the gene), but absent in the NS-ΔICL mutant

(lanes 3 and 4).

(D) Assessment of genetic stability after 30 generations of serial culture. The *NsICL* gene locus was checked again to ensure the genetic stability of the mutations. The deletion mutant (NS-ΔICL, lane 1) stably lacked the band, while the complemented strain (NS-cΔICL, lane 2) and wild-type (lane 3) retained it, confirming that the ΔICL mutation is stable and not revertible.

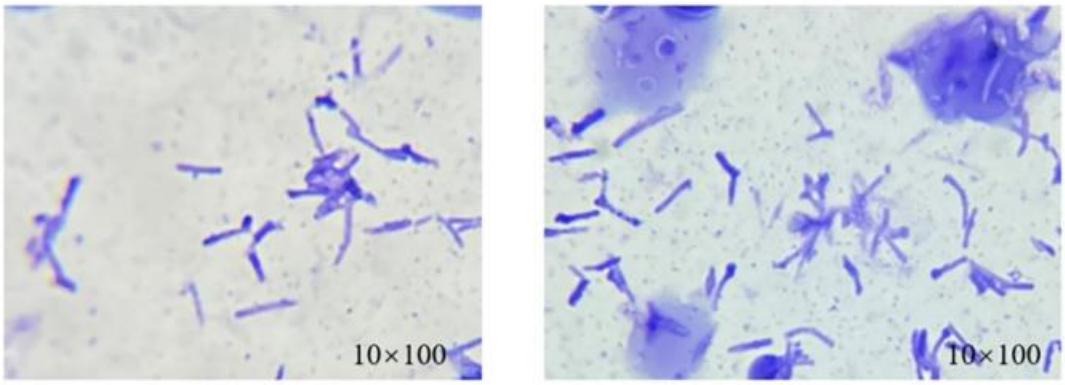

Figure 7 Hyphae of the deletion strain NS-ΔICL (left) and wild-type strain ZJ0503 (right)

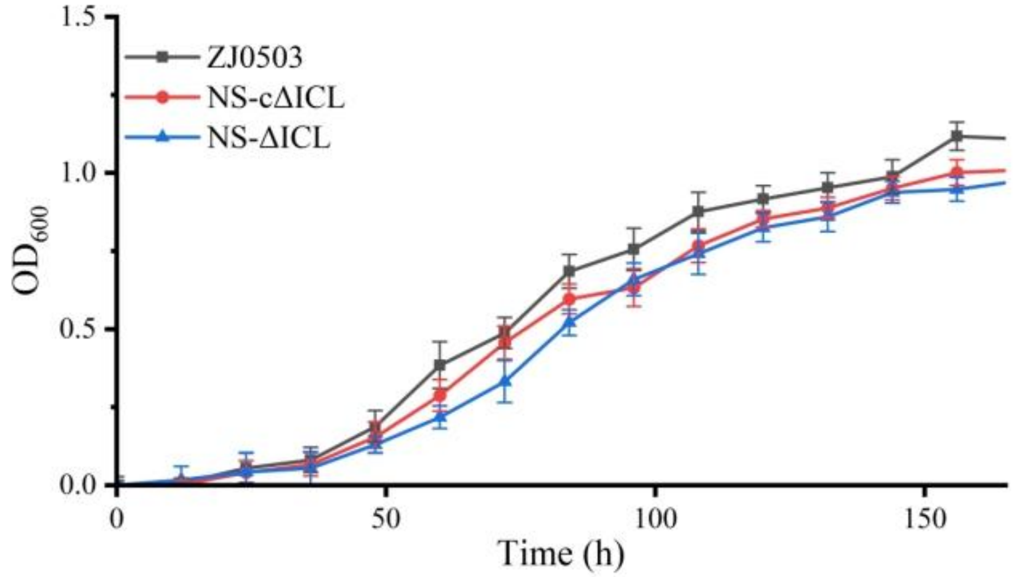

Figure 8 The growth curve of N. seriolae strain NS-ΔICL, NS-cΔICL and ZJ0503. Data are presented as mean ± standard deviation (SD). N = 3 represents the number of biological replicates (three independent bacterial cultures), with three technical replicates (three OD measurements per bacterial culture) performed for each biological sample.

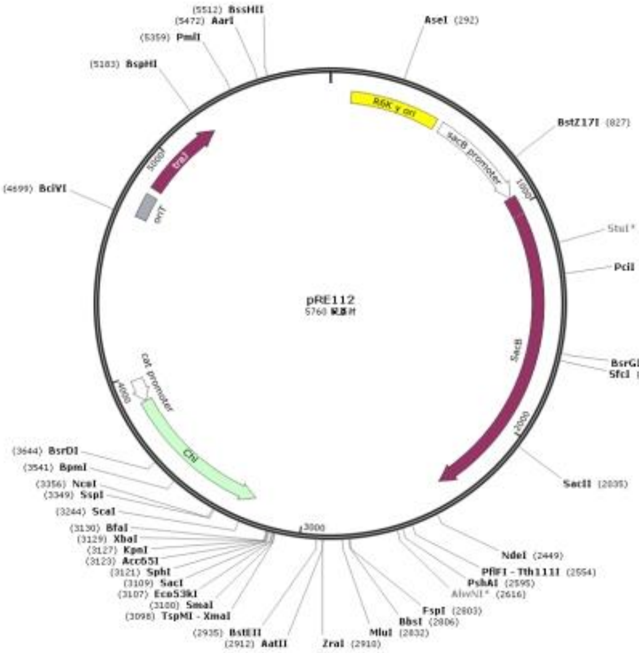

Figure 9 Map of the pRE112 plasmid
