# Supplementary material for: An isocitrate lyase gene-deleted strain of Nocardia seriolae in live attenuated vaccine development against fish nocardiosis
Source: Front Vet Sci. 2025 Oct 31;12:1664034. doi: 10.3389/fvets.2025.1664034 (PMC12615200; doi:10.3389/fvets.2025.1664034)
Supplement: Supplementary file 2 [file Data_Sheet_2.pdf]

Table S1

Primers used for establishing the deletion strain NS-ΔICL.

Name	Sequence (5′ -3′)	Aim
ICL-UF	CCGACGCGTGCCTCTTTCGGCTGAAATC	Plasmid construction, amplify the upstream of *NsICL*（506bp）
ICL-UR	CTTCGGGTGGCCTCTGACTCCACTTCTTCG	
ICL-DF	GTGGAGTCAGAGGCCACCCGAAGCGGCTCGG	Plasmid construction, amplify the downstream of *NsICL*（506bp）
ICL-DR	TAGTCTAGAGCTCGCGCTCGCGCTCGGTG	
112-F	ATCTTGCGAATATATGTGTAGA	Checkout the pRE112
112-R	TAACCAGACCGTTCAGCTG	
ICL-F2	GGTGCCGACGTTCGACAT	Detect the positive clone of NS-ΔICL
ICL-R2	ATGTCGAACGTCGGCACC	

Note: The straight line is the overlapping PCR site and the underline is the enzyme site.
