# Supplementary material for: An isocitrate lyase gene-deleted strain of Nocardia seriolae in live attenuated vaccine development against fish nocardiosis
Source: Front Vet Sci. 2025 Oct 31;12:1664034. doi: 10.3389/fvets.2025.1664034 (PMC12615200; doi:10.3389/fvets.2025.1664034)
Supplement: Supplementary file 3 [file Data_Sheet_3.pdf]

Table S2

The confidence degree was calculated for the LD_{50} of NS-ΔICL

	95% CI of inoculum			95% CI of log(inoculum) [a]		
Probability	Estimates	Inferior limit	Superior limit	Estimates	Inferior limit	Superior limit
.010	9.313	.010	193.375	.969	-2.015	2.286
.020	44.617	.127	604.805	1.649	-.897	2.782
.030	112.642	.579	1190.452	2.052	-.237	3.076
.040	218.803	1.716	1938.409	2.340	.235	3.287
.050	368.189	4.016	2844.947	2.566	.604	3.454
.060	565.850	8.095	3910.305	2.753	.908	3.592
.070	816.934	14.719	5137.179	2.912	1.168	3.711
.080	1126.770	24.821	6530.047	3.052	1.395	3.815
.090	1500.935	39.518	8094.834	3.176	1.597	3.908
.100	1945.301	60.134	9838.751	3.289	1.779	3.993
**.150**	**5452.136**	**315.676**	**21582.345**	**3.737**	**2.499**	**4.334**
.200	11845.327	1082.651	39617.245	4.074	3.034	4.598
.250	22484.032	2945.944	66543.461	4.352	3.469	4.823
.300	39356.486	6927.530	106728.193	4.595	3.841	5.028
.350	65441.371	14714.990	167777.702	4.816	4.168	5.225
.400	105312.866	28942.964	263572.912	5.022	4.462	5.421
.450	166194.363	53492.765	420685.220	5.221	4.728	5.624
**.500**	**259873.217**	**93818.914**	**692325.629**	**5.415**	**4.972**	**5.840**
.550	406356.074	157553.231	1189931.636	5.609	5.197	6.076
.600	641271.016	256093.067	2159533.913	5.807	5.408	6.334
.650	1031978.511	408426.471	4184017.872	6.014	5.611	6.622
.700	1715958.310	649671.263	8783152.200	6.235	5.813	6.944
.750	3003646.735	1051394.268	20469451.500	6.478	6.022	7.311
.800	5701327.723	1778003.699	55321770.634	6.756	6.250	7.743
.850	12386721.501	3280699.294	188753543.476	7.093	6.516	8.276

.900	34716519.948	7225788.397	986864598.773	7.541	6.859	8.994
.910	44994670.332	8788745.268	1500607264.705	7.653	6.944	9.176
.920	59935990.506	10902284.030	2387527461.582	7.778	7.038	9.378
.930	82667772.423	13867526.608	4023419021.204	7.917	7.142	9.605
.940	119349801.342	18230391.871	7310769397.707	8.077	7.261	9.864
.950	183422443.816	25073265.188	14725795295.595	8.263	7.399	10.168
.960	308653054.121	36822810.658	34442665716.205	8.489	7.566	10.537
.970	599547420.188	59997294.961	102049689953.788	8.778	7.778	11.009
.980	1513653970.464	118174667.620	465698595726.110	9.180	8.073	11.668
.990	7251971360.589	369893519.358	6108809733827.600	9.860	8.568	12.786

Note. Logarithmic base=10
