# Supplementary material for: An isocitrate lyase gene-deleted strain of Nocardia seriolae in live attenuated vaccine development against fish nocardiosis
Source: Front Vet Sci. 2025 Oct 31;12:1664034. doi: 10.3389/fvets.2025.1664034 (PMC12615200; doi:10.3389/fvets.2025.1664034)
Supplement: Supplementary file 4 [file Data_Sheet_4.pdf]

Table S3

The confidence degree was calculated for the LD_{50} of *N. seriolae* ZJ0503

	95% CI of inoculum			95% CI of log(inoculum)		
Probability	Estimates	Inferior limit	Superior limit	Estimates	Inferior limit	Superior limit
.010	13.359	.045	175.334	1.126	-1.351	2.244
.020	46.023	.330	434.139	1.663	-.481	2.638
.030	95.614	1.075	743.696	1.981	.032	2.871
.040	161.503	2.504	1095.520	2.208	.399	3.040
.050	243.570	4.852	1485.731	2.387	.686	3.172
.060	341.958	8.371	1912.358	2.534	.923	3.282
.070	456.971	13.332	2374.444	2.660	1.125	3.376
.080	589.038	20.025	2871.665	2.770	1.302	3.458
.090	738.685	28.766	3404.129	2.868	1.459	3.532
.100	906.522	39.897	3972.272	2.957	1.601	3.599
.150	2045.267	145.498	7380.103	3.311	2.163	3.868
.200	3773.995	381.999	11870.487	3.577	2.582	4.074
.250	6259.577	840.482	17730.558	3.797	2.925	4.249
.300	9738.893	1658.359	25407.981	3.989	3.220	4.405
.350	14550.007	3042.320	35598.386	4.163	3.483	4.551
.400	21183.617	5301.692	49411.890	4.326	3.724	4.694
.450	30369.017	8895.901	68690.587	4.482	3.949	4.837
**.500**	**43222.660**	**14500.543**	**96635.630**	**4.636**	**4.161**	**4.985**
.550	61516.590	23100.560	139102.017	4.789	4.364	5.143
.600	88190.718	36136.998	207417.332	4.945	4.558	5.317
.650	128398.456	55793.190	324957.633	5.109	4.747	5.512
.700	191828.622	85658.128	544033.703	5.283	4.933	5.736
.750	298454.401	132380.183	995335.416	5.475	5.122	5.998
.800	495018.820	210115.514	2060886.416	5.695	5.322	6.314

.850	913425.397	354704.346	5155327.246	5.961	5.550	6.712
.900	2060840.963	685138.477	18083603.149	6.314	5.836	7.257
.910	2529086.155	805036.019	24907968.079	6.403	5.906	7.396
.920	3171607.017	960717.660	35542167.050	6.501	5.983	7.551
.930	4088218.744	1169486.145	53040160.339	6.612	6.068	7.725
.940	5463242.047	1461345.035	83933434.068	6.737	6.165	7.924
.950	7670053.870	1892810.393	143901159.242	6.885	6.277	8.158
.960	11567601.253	2583127.700	277097436.879	7.063	6.412	8.443
.970	19539010.584	3829440.161	641174062.889	7.291	6.583	8.807
.980	40592733.746	6604088.405	2074895013.829	7.608	6.820	9.317
.990	139848630.612	16477888.091	15245369001.894	8.146	7.217	10.183

Note. Logarithmic base=10
